# The Effect of Ecological Management on Regional Health Inequality

**DOI:** 10.3390/ijerph20043037

**Published:** 2023-02-09

**Authors:** Fafa Yan, Alec Zuo, Wen’e Qi, Zhimin Zhou

**Affiliations:** 1 Lingnan College, Sun Yat-Sen University, Guangzhou 510275, China; 2 Centre for Global Food and Resources, School of Economics and Public Policy, University of Adelaide, Adelaide, SA 5000, Australia; 3 College of Economics and Management, South China Agricultural University, Guangzhou 510642, China; 4 School of Finance and Economics, Guangdong Polytechnic Normal University, Guangzhou 510665, China

**Keywords:** health inequality, China, ecological management, causality, endogeneity, sys-GMM

## Abstract

Ecological management has been implemented to improve individual well-being. However, it remains unclear whether this management has improved health inequality over time. Aiming to examine whether health inequality is caused by ecological management in China, we harnessed a macro-level dataset from 2001 to 2019 across 31 Chinese provinces—combined with gene and dietary culture data—and utilized a bilateral approach to pair provincial data. Empirical results of system Generalized Method of Moments (sys-GMM) estimations in benchmark and extensive models which suggest a negative and statistically significant causal effect of ecological management on health inequality. Specifically, ecological management contributes to decreasing the inequality in the population death rate, the death rate among pregnant women, the underweight newborn rate, the child malnutrition rate, and the infectious disease mortality. The results are robust to weak instruments in the sys-GMM setting and a delayed effect of ecological management. Additionally, the heterogeneity analysis shows that the causal effect of ecological management on decreasing regional health inequality is more significant and higher for subsamples in identical regions than in different regions.

## 1. Introduction

Since the reform and opening-up policy (ROP) three decades ago, urbanization in China has experienced rapid development [1,2], contributing to social advancement and substantial economic growth [3]. Meanwhile, urbanization causes various side effects, significantly worsens ecological circumstances, and increases pollution [1,4,5,6]—resulting in severe ecological and environmental consequences in China [7] and even around the world [8]. A large segment of the population opposed sacrificing the ecological environment for urbanization and economic development [1,9].

Thus, China instituted a series of policies on ecological management to alleviate these issues and guarantee a sustainable development during four stages, including End-of-pipe Governance (1980s–1990s), Cleaner Production (1990s–2000s), Circular Economy (2000s–2010s), Ecological Civilization (2010s–present) [10]. Maintaining a sound ecological environment is a universal human well-being benefit [11]. The ecological environment is closely related to each individual. Ecological management must be people oriented and focused on solving prominent environmental problems that harm people’s health [12].

Furthermore, many problems involved in ecological environment were found to be intertwined, which led to significant gaps between the expected quality of the ecological environment and the actual quality of the ecological environment [7]. Therefore, the current primary task is to improve the actual quality of the ecological environment, enhance the government’s management of the ecological environment, and overcome the deficiencies within the ecological environment [12].

Nevertheless, due to significant improvement in living standards brought about by the ROP, Chinese people have increasingly demanded an improved ecological environment. They are paying more attention to the actual quality of the ecological environment and health issues [7]. The overall requirements for promoting ecological management have essential theoretical and practical significance [7,13].

Our overall research aim is to explore the negative causal correlation between ecological management and regional health inequality at the macro background where China implemented mandatory policies to address the issues caused by the ecological environment. Specifically, the study (1) constructs a composite index of ecological management by using the entropy weight method; (2) sets up a baseline model under the assumption that government strives to improve population health levels, yet individuals make identical efforts, that is to say, they do not make any efforts to improve their health status; (3) extends the baseline model by relaxing the assumption that both government and individuals make efforts to improve individual health; (4) carries out a sys-GMM estimate for the baseline and extended models, to verify that ecological management is negatively related to regional health inequality and identify the causality from ecological management to regional health inequality; and (5) conducts a heterogeneity analysis to explore the various causal effects of the ecological management on regional health inequality in China.

The present study contributes to the literature in four aspects. First, while literature exists on overall health inequalities, disaggregated measures for specific demographic groups such as the elderly, children, and women are scarce. We fill this gap by studying the death rate among pregnant women, the underweight newborn rate, the child malnutrition rate, and infectious disease mortality. Second, most of the existing literature focuses closely on measuring health inequality, based on population, wealth, and other factors—and thus cannot measure pure health inequalities. We employ measures of local differences in the regional economic domain that can describe pure health inequalities. Third, while there is extensive literature on the environment, lifestyle, and healthcare organizations affecting health inequality, no previous studies have analyzed the effects of ecological management on health inequality. This study constructs a composite index of ecological management as defined by China’s policies and bridges the gap in the causal relationship between ecological management and regional health inequality. Finally, this study incorporates ecological management into the Health Field Concept model and the Effort–Circumstances theory; empirically confirming the impact of ecological management on regional health inequality; and extends the application of the conceptual model to the health field.

This study combines qualitative and quantitative analysis to identify the causal correlation between ecological management and regional health inequality. Specifically, the methodology includes (1) a qualitative analysis, a theoretical extension by adding ecological management into our theoretical framework which is merged by both the Health Field Concept model and the Effort–Circumstances theory, (2) a quantitative analysis based on the theoretical extension. Ecological management is measured by the entropy weight method; regional health inequality is calculated by the bilateral approach. We leveraged a sys-GMM to estimate the coefficients because sys-GMM is an appropriate approach to deal with the potential endogeneity of the ecological management and identifies a correct causal relationship.

## 2. Literature Review

### 2.1. Health Inequality

Many studies have investigated the nexus between the key determinants of health and health inequality [14,15,16,17,18,19,20,21,22,23,24,25,26,27,28,29,30,31,32,33,34,35,36,37]. Health Field Concept theory—a widely accepted conceptual framework—envisages the health field being subdivided into four principal elements, i.e., human biology, environment, lifestyle, and healthcare organization [14]. According to the Health Field Concept theory, the most relevant studies are divided into two branches. One branch was involved in the environment and lifestyle elements. For example, Zhou et al. [15] focused on income-related health inequality, such as household income and consumption expenditure in China. Davillas and Jones [16] identified that socioeconomic circumstances, i.e., education and childhood socioeconomic status, could decrease health inequality—while relative discrepancies in age and gender could increase health inequality for the samples with higher health risk in the UK. Azimi et al. [17] considered education, income inequality, secondary industry, and urbanization as measures of socioeconomic status (SES) and indicators of health inequality in China. Mazeikaite et al. [18] identified the determinants of health inequality among European nations, which included education, labor market status, and income in the EU. Chen et al. [19] found that income growth and accessibility to public transport contributed to addressing inequality in terms of child malnutrition in China. Cavalieri and Ferrante [20] found that a province’s population is significantly related to regional health inequality in Italia. Fang et al. [21] revealed that SES is significantly associated with regional health inequality across China.

Additionally, the other branch of the most relevant literature discussed the impacts of healthcare organization on health inequality. Fang et al. [21] identified that greater overall health resources produced lower health differentials in China. Azimi et al. [17] found inequality in health expenditure, green space in cities, and the number of hospital beds as key indicators of health resources—highly significant for health inequality in China. Furthermore, many studies acknowledged access to health services as a strong influence on health inequality: Liu et al. [22] found that health services were likely to make a significant difference in terms of health inequality in Rwanda; Fang et al. [21] discovered that the utilization ratio of hospital beds is vital to reducing health inequality in China; while Cavalieri and Ferrante [20] revealed that the provincial out-patient rate in hospitals positively impacted regional health in Italia.

In summary, studies have focused on the impacts of circumstances (family environment, residing environment, and geographic environment) on regional health inequality at the macro and micro levels. Due to the lack of relevant data, most studies assume that all individuals’ biological traits and lifestyles are in common with one another. However, according to the Effort–Circumstances theory, an individual’s health status is caused by two factors, i.e., the individual’s effort and the circumstances he/she stays in [23]. Moreover, the health inequality resulting from circumstance is defined as ‘health opportunity inequality’ or ‘unfair health inequality’ and that from an individual’s efforts, such as lifestyle, and health literacy [23] is termed as ‘reasonable health inequality’ or ‘fair health inequality’ [24]. Therefore, this theory emphasizes that individuals’ efforts to take responsibility for their health status are not responsible for decreasing health inequality; it is circumstances that play a role in bridging the health gap. This study discriminates between ‘unfair health inequality’ and ‘fair health inequality’. It proposes that apart from individuals’ efforts (‘fair health inequality’), the central government (‘unfair health inequality’) also has an opportunity to reduce regional health inequality and focuses on the government’s effort to improve individuals’ circumstances (‘unfair health inequality’).

Overall, regardless of whether individuals could or could not make efforts to improve their health status, they could not change their environment. However, the government could also improve the macro circumstances where individuals live, work, and entertain by managing the nationwide or regional ecological environment. It is difficult for the government to change individuals’ biological characteristics, lifestyle factors, healthcare organization, and other environmental factors such as family environment, community environment, and geographical environment in the short run at least, and hence the government’s effort to improve individuals’ health status and alleviate the regional health inequality primarily through ecological management. Thus, effort in this study is divided into two parts. One is for individuals to improve their health status directly, in the form of lifestyle and education, and the other is for government to improve individuals’ health status indirectly, in the form of ecological management. Several scholars have focused on how individuals’ efforts affect health inequality [16,25]. Nevertheless, there is still a significant gap regarding the nexus of the government’s effort, i.e., ecological management, and regional health inequality, to investigate.

Using selected indicators, ecological management, human biology (blood type *ABO* genes and immunoglobulin allotypes Gm gene), environmental factors (family, residing and geographical environment), and healthcare organization (medical resources and services), we aim to explore whether the government’s effort (ecological management) could decrease regional health inequality when we assume that all the individuals have an identical lifestyle. Then, we relax the assumption and add lifestyle factors, including 11 dummies of dietary culture circles, i.e., Jingjin, Dongbei, Zhongbei, Xibei, Huanghezhong, Huanghexia, Changjiangzhong, Changjiangxia, Dongnan, Xinan, Qingzang, and education inequality, and aim to further identify whether ecological management could decrease regional health inequality if the individuals have discrepant lifestyles. A comparison of their results among the five health indicators allows us to find when government and individuals are responsible for decreasing health inequality.

Health inequalities are dimensional concepts based on measurable indicators of evaluating health status, and therefore, selecting appropriate indicators for health status is essential before measuring health inequality. Generally, two major approaches have been described for evaluating health status: the first method involves mortality rate [17,26,27], the morbidity rate of health items [28,29]—focusing on child growth, the health of women, and health of the overall population. The second method introduces life span [26,30,31,32] or individual self-reported health (SRH) [33,34]. The most effective way to select health indicators is to determine the most appropriate for research purposes, methods, and perspectives—while deciding on the micro or macro level.

A branch of studies has focused on measuring health inequality [31,35,36], identifying two widely accepted types of measuring regional health inequality. These are the principal component analysis (PCA) for multiple dimensions of health indicators [21] and the traditional inequality index—such as the Lorenz curve and Gini coefficient [37]; Concentration Index [15,34]; and Theil Index [20,30]. This study uses a bilateral approach to calculate relative regional health inequality. The main reasons are threefold: First, the measurement is mainly determined by the health outcomes of interest and the availability of their datasets [38]. We are interested in province pair health inequality; thus, the bilateral approach is a suitable choice. Second, it is an approach commonly applied to analyze regional inequality [39,40,41,42] and could be employed to measure regional health in-equality. Third, the PCA method involves a cohort of variables that would lose part of their information during the analysis. Additionally, the regional inequality index method does not avoid referencing population or wealth, which produces a disadvantage—we could not measure pure health inequality effectively. Conversely, measuring health inequality through a bilateral approach could sufficiently deal with the abovementioned problems.

### 2.2. Ecological Management and Health Effects

The level of ecological management and its evaluation is one of the most vexing is-sues in the ecological economics domain. For example, Li et al. [9] quantified ecological management in China, but focused only on Lianyungang city. Yao et al. [43] calculated the ecological efficiency of 30 provinces across China using the super-SBM model. Based on the above considerations, this article aims to build an ecological management index that can fully reflect the new Chinese era, based on the key goal of ecological civilization construction. However, it is subject to the overall level of China’s ecological management according to this index. In this study, we also analyze the spatial and temporal evolution of each index.

However, current studies mainly focus on the measurement of ecological management. Governance, including the authorities’ ecological management, has been identified as a critical factor in child health [44]. Although it is only one component of overall governance, ecological management is highly likely to influence health and health inequality. Furthermore, ecological management can be viewed as the effort made by the Chinese government to improve individuals’ health status. Ecological management is an integral part of national governance and ecological management was listed in the national policies and plans. The Central Committee and The State Council in China have attached great importance to ecological and environmental protection. Since the 12th Five-Year Plan, the Communist Party of China (CPC) has resolutely declared a fight against pollution; made significant efforts to prevent and control air, water, and soil pollution; continued to intensify efforts to protect ecology and the environment; improved ecological and environmental quality; and accomplished the main targets and tasks set out in the 12th Five-Year Plan. During the 13th Five-Year Plan, the problems of imbalance, discoordination, and unsustainability in economic and social development are still prominent. Accordingly, in China, ecological management involves significant policies and plans. The 18th National Congress of the CPC included the construction of an ecological civilization within China’s ‘five-in-one’ overarching strategy. Giving top priority to ecological management is a requirement under the national Chinese strategy to improve individual livelihoods and best meet their living needs. It has identical importance to individuals’ efforts based on the Effort–Circumstances theory [23].

**Figure 1:**
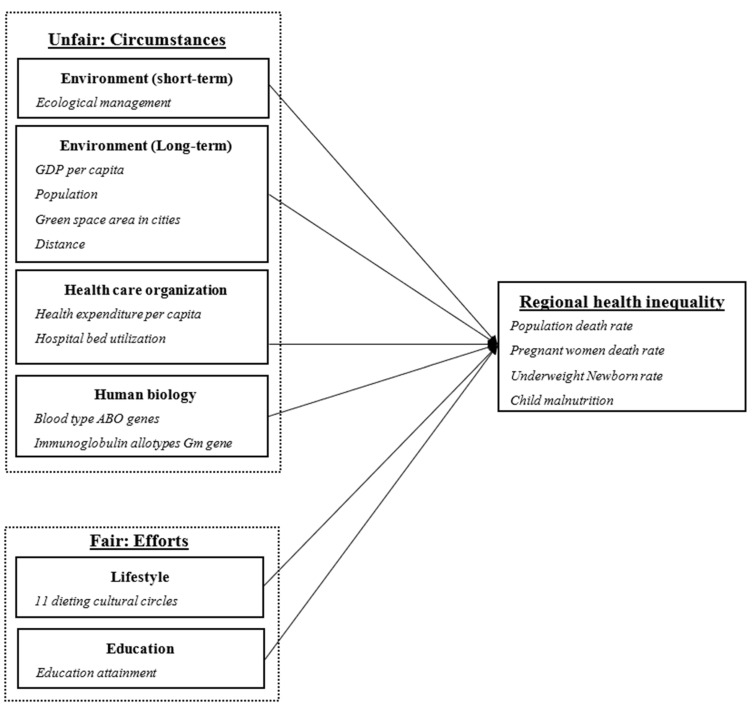
The conceptual framework of the impact of ecological management on regional health inequality. Source: The Health Field Concept model by Lalonde [14] and the Effort–Circumstances theory by Roemer [23].

Therefore, based on the Health Field Concept model and the Effort–Circumstances theory, this study constructs a conceptual framework to investigate how ecological management influences regional health inequality. The conceptual framework is demonstrated in Figure 1.

The remaining parts of the study are organized as follows: Section 2 reviews the literature on health inequality, ecological management, and health effects. Section 3 describes the variable selection, descriptive statistics, and data sources. Section 4 introduces the methodology. Section 5 reports the empirical results of sys-GMM estimations for the baseline model and extended model, to examine the causal effect of ecological management on regional health inequality and heterogeneous analysis, as well as discussions. Section 6 outlines the conclusion and the policy implications.

## 3. Variables, Descriptive Statistics, and Data Sources

### 3.1. Variables

#### 3.1.1. Dependent Variable: Health Inequality

The health indicators comprise five variables, namely, population death rate (unit: ‰), pregnant women death rate (unit: per 100,000 persons), underweight newborn rate (rate of newborn babies with a birth weight under 2500 g, unit: %), child malnutrition rate (rate of children under five years old with severe malnutrition, unit: %), and infectious disease mortality (unit: per 100,000 persons). Regional health inequality is defined as the absolute value of the difference between logarithmic health indicator $H_{ijt}$ in province *i* and logarithmic health indicator *j* $H_{kjt}$ in province *k*, which is known as a bilateral approach [40,41,42,45] and noted as:

$$Hineq_{ikjt}=|ln(H_{ijt})-ln(H_{kjt})|$$

where i, k, and t denote the province, health indicator j, including five health indicators.

This definition is similar to differences or inequality among economic variables within the regional economic literature [39,41,42,43].

#### 3.1.2. Key Variable: Ecological Management 

A group of elements could determine regional health inequality and a critical one is ecological management. Ecological management is an important part of Chinese national governance. The fundamental goal of promoting reform and development is to meet the basic living needs of the population. These include a variety of material and cultural needs along with ecological environment needs. The definition of ecological management is calculated by six indicators, because the goal of China’s policies for ecological management is to achieve resource savings, environmental friendliness, resource recycling, a sharp drop in carbon dioxide emissions, a significant reduction in main pollutant emissions, as well as a significant improvement in forest coverage and ecosystem stability. According to the overall requirements of the above objectives and the literature review by Guo et al. [12], the ecological management index system constructed in this paper covers six indicators: water environment, air environment, pollution treatment, green environment, residential environment, and soil environment. $Ecogovern_{ikt\;}$ denotes the overall ecological management level between provinces i and k in year t, a composite index calculated using the entropy weight method by Guo et al. [12], and Chen and Shi [46] on the most relevant indicators related to ecological management.

Based on the theoretical deduction and empirical observation from the literature review by Guo et al. [12], Chen and Shi [46], and Brand and Missaoui [47], we identified six indicators of ecological management with a total of 30 items that are used to construct our overall ecological management index. The six indicators are water (eight items), air (four items), pollution treatment (six items), the green environment (five items), the residential environment (four items), and soil (three items) (see Table 1 for more detailed information).

The entropy weight method is an approach to measuring a composite index based on a range of indicators. As an objective method to calculate a composite index, it calculates the weight according to all information within every single indicator to the composite index. The method of entropy weight to evaluate a composite index is shown in the study of Feng et al. [48].

We first employed the entropy weight method to calculate every single indicator for the six indicators by employing the corresponding items (see Column 2 in Table 1), respectively; and then we computed the composite index of ecological management by using the six indicators together items (see Column 1 in Table 1).

#### 3.1.3. Control Variables 

Based on the framework developed by Lalonde [14], the provincial control variables include human biology, environment, lifestyle, and healthcare organization. The detailed definitions of the control variables are shown as follows.

The first indicator is human biology. Nei’s genetic distance [49] between two provinces includes two proxy variables. The first variable is Nei’s genetic distance blood group, defined as Nei’s gene distance of blood type ABO genes between province pairs. The calculation and gene frequency originated from Chen et al. [50], which provided the frequency of the three genes across 27 inland provinces, along with missing data from Beijing, Tianjin, Shanghai, and Chongqing. Among them, the gene distance from Beijing and Tianjin to the other provinces was supplemented with that from Hebei since Hebei is their nearest province.

Similarly, the missing data for Shanghai was supplemented by the mean of Jiangsu and Zhejiang, and Sichuan supplemented the gene distance in Chongqing. According to genetic distance and cluster analysis, blood group distribution in China is divided into four groups: 1 = *B* gene, including Hainan and provinces in North and Northwest China; 2 = *A* gene, including Yangtze River basin and southwest; 3 = *O* gene, including Southeast China, and 4 = others, i.e., Tibet. The second variable is the Nei’s distance between the immunoglobulin allotype gene 2, defined as the Nei’s gene distance of immunoglobulin allotypes Gm gene between province *i* and province *k*. The frequency of Gm genes originated from Zhao et al. [51], which provided genetic data from 74 provinces and municipalities. Data from different sampling points in the same province were directly combined. Missing data in Beijing and Tianjin were supplemented using the nearest province, Hebei.

The second indicator is environmental factors. Environmental factors chiefly include family environment, residing environment and geographical environment. The family environment mainly considers the economic environment, using the residents’ income level as a proxy variable [20,24], which is defined as the absolute value of the difference in the natural logarithm of the annual per capita real GDP (¥ per capita, adjusted based on the CPI in 2001) between province pair, recorded as $RGDP_{ikt}$:

$$RGDP_{ikt}=|ln(RGDP_{it})-ln(RGDP_{kt})|$$

The living environment is mainly the gap in population size $POP_{ikt}$ [20] and the green environment of the provinces $GSA_{ikt}$ [17], measured by the absolute value of the difference between the natural logarithm of the end of the permanent resident population (10,000 persons) and the difference between the natural logarithm of the green space in the city (10,000 square meters), namely:

$$POR_{ikt}=|ln(POR_{it})-ln(POR_{kt})|$$

$$GSA_{ikt}=|ln(GSA_{it})-ln(GSA_{kt})|$$

Among them, the provinces with an urban green space area of 0 should add 0.001 to the supplement, to ensure that the logarithmic operation is meaningful. The geographic environment is mainly geographical distance, region, and boundary. The geographical distance is measured by the natural logarithm of geographical distance $Dist_{ik}$ between province *i* and province *k* (km). Regional variables $region_{ik,m}$ denoted, respectively: (1) if both the paired provinces *i* and *k* belong to Central China, $region_{ik,1}=1$ otherwise $region_{ik,1}=0$; (2) if both the paired provinces *i* and *k* belong to Eastern China $region_{ik,2}=1$, otherwise $region_{ik,2}=0$; (3) if both the paired provinces *i* and *k* belong to Western China, $region_{ik,3}=1$ otherwise $region_{ik,3}=0$; (4) if the paired provinces *i* and *k* belong to Eastern or Central China, $region_{ik,4}=1$, otherwise $region_{ik,4}=0$, respectively; (5) if the paired provinces *i* and *k* belong to Eastern or Western China, $region_{ik,5}=1$ otherwise $region_{ik,5}=0$, respectively. The base group is whether paired provinces belong to Central and Western China, respectively. The boundary variable $Border_{ik}$ is a dummy variable. If province *i* borders province *k*, it implies that consumers in adjacent provinces tend to have interprovincial health consumption behavior. The cost of health arbitrage is lower and much more likely to be less than the benefits of such arbitrage. There is no boundary isolation effect; if the provinces *i* and *k* are not geographically adjacent, there is a boundary isolation effect between the two provinces.

Third indicator is lifestyle factors. These refer to individual efforts that can sway their health inequality. The most crucial embodiment is the dietary habits and inequality in education level. It is not easy to depict an eating diet at the macro level. According to the seminal work by Zhao [52], Chinese Food Culture History, China’s dietary culture is divided into eleven circles: 1 = Northeast, 2 = Beijing and Tianjin, 3 = North, 4 = Northwest, 5 = downstream Yellow River, 6 = midstream Yellow River, 7 = midstream Yangtze River, 8 = downstream Yangtze River, 9 = Southeast, 10 = Southwest, 11= Qinghai-Tibet Plateau. A variable is generated to describe if the paired provinces belong to the same dietary culture circle $diet_{iks}=1$ $diet_{iks}=0$ s=1,⋯,11. Inequality in education (EDU) is always seen as a further lifestyle indicator [20], and theoretical analysis reveals that individuals with higher education levels tend to live longer [30]. Accordingly, we select inequality in average education attainment $EDU_{ikt}$ to describe the years of average education attainment between the paired provinces in China as a proxy of education inequality, as defined below:

$$EDU_{ikt}=EDU_{it}÷EDU_{kt}$$

where $EDU_{it}$, $EDU_{kt}$ denotes average education attainment in province i and *k* in the year *t*, respectively.

The fourth indicator is healthcare organization. This embraces medical resources and services. The inequality in medical resources between the paired provinces, measured by the gap in healthcare expenditure $EXPEN_{ikt}$, refers to the inequality in per capita healthcare expenditure (¥, adjusted based on CPI in 2001), i.e.,

$$EXPEN_{ikjt}=|ln(EXPEN_{ijt})-ln(EXPEN_{kjt})|$$

The per capita healthcare expenditure of urban and rural residents in some provinces in year *t* equals the sum of per capita healthcare expenditure of urban residents per capita healthcare expenditure. The gap in hospital bed utilization characterizes the healthcare service gap and patient outflow rate between the two paired provinces, defined as follows, respectively:

$$URB_{ikt}=URB_{it}÷URB_{kt}$$

where $URB_{it}$ and $URB_{kt}$ stand for bed utilization proportion in hospitals in province *i* and *k* in year *t*, respectively.

$$POR_{ikt}=POR_{it}÷POR_{kt}$$

where $POR_{it}$ and $POR_{kt}$ are patient outflow rate in province *i* and *k* in year *t*, respectively; $POR_{it}$ is calculated by the number of discharged patients divided by the total number of hospital admissions.

The definitions and descriptive statistics of the dependent, key, and control variables are listed in Table 2.

### 3.2. Data Sources

The data for the ecological management indicators in 31 provinces (excluding Taiwan, Hongkong and Macao) from 2001 to 2019 are from the website of the China National Bureau of Statistics [53]. The health data were sourced from the Chinese Health and Family Planning Statistical Yearbook (2002–2020). The data for constructing the development level and development difference variables of ecological management originate from the official website of the Bureau of Statistics. For the control variables, the biological and genetic data were obtained from Chen et al. [50] and Zhao et al. [51], including research results, environmental factors, and healthcare systems. Each variable dataset was obtained from the National Bureau of Statistics and China Health and Family Planning Statistical Yearbook (2002–2020); lifestyle variable data were sourced from the National Bureau of Statistics and Zhao [52]—the Chinese Food Culture History.

## 4. Methodology

### 4.1. Baseline Model: The Assumption of an Identical Lifestyle for All the Individuals

In the baseline model, we aim to explore what the government could do to address health inequality, assuming individuals do not take any steps to improve their health status—leading to a further assumption that all individuals have an identical lifestyle. Hence, we do not incorporate control variables for lifestyle. The baseline model is specified as follows:

(1) $$Hineq_{ikjt}=\beta_{0}+Hineq_{ikjt-1}+\beta_{1}Ecomanage_{ikjt}+{X^{\prime }}_{ikjt}\gamma +u_{ikj}+v_{jt}+\varepsilon_{ikjt}$$

where $Hineq_{ikjt}$ characterizes the regional health inequality in province pairs (province *i* and province *k*) in year *t*, $Ecomanage_{ikjt}$ is the key variable in this study, capturing the overall level of ecological management. $\beta_{1}$ is representative of the marginal effect of ecological management on health inequality. $X_{ik}$ denotes the control variable, including human biology, environment, healthcare organization, and time effects; more details are reported in Table 2. $u_{ik}$ is the province pair’s random effect, $v_{t}$ depicts time effect, while $\varepsilon_{ikt}$ indicates the random error term.

### 4.2. Extended Model: Relax the Assumption of an Identical Lifestyle for All the Individuals

In the extended model, we relax the assumption of an identical lifestyle and add the control variables of the lifestyle so that we can investigate how the government’s effort to improve population health status and diminish health inequality when individuals also make efforts to improve their health status. The extended model is established as follows:

(2) $$Hineq_{ikjt}=\beta_{0}+Hineq_{ikjt-1}+\beta_{1}Ecomanage_{ikjt}+\beta_{2}Effort_{ikjt}+{Z^{\prime }}_{ikjt}\gamma +u_{ikj}+v_{jt}+\varepsilon_{ikjt}$$

where $Z_{ik}$ denotes the control variable, including human biology, environment, and healthcare organization, and the definition of human biology, environment, healthcare organization in Equation (2) are the same as that in Equation (1). Effort incorporates lifestyle and education attainment (see Table 2 for more detail).

### 4.3. Sys-GMM Estimation

In this subsection, we examine the impact of ecological management on health inequality in China, assuming that individuals do not make any efforts to improve their health levels. Estimating fixed effects for dynamic panel data is inherently biased [54]; hence, sys-GMM is an efficient and appropriate method [55]. Furthermore, the unavoidable error of measuring ecological management tends to induce potential endogeneity problems and may produce inconsistent estimation and erroneous inference. Thus, following the methodologies [56], we can leverage the sys-GMM method to address the endogeneity problem of ecological management.

We combine the differential GMM and level GMM, and estimate them as a system developed by Blundell and Bond [55]. The differential form of Equation (1) is set up as follows:

(3) $$\Delta Hineq_{ikjt}=\Delta Hineq_{ikjt-1}+\beta_{1}\Delta Ecogovern_{ikjt}+\Delta {X^{\prime }}_{ikjt}\gamma +\Delta v_{jt}+\Delta \varepsilon_{ikjt}$$

where $\Delta Hineq_{ikjt}=Hineq_{ikjt}-Hineq_{ikjt-1}$, and the Δ operation applies to other variables too.

The sys-GMM assumes that the disturbance term $\varepsilon_{ikjt}$ does not auto-correlate, i.e., $cov(\varepsilon_{ikjt},\varepsilon_{ikjs})=0,t\neq s,\forall ik$ and {$\Delta Hineq_{ikjt-1}$, $\Delta Hineq_{ikjt-1}$,…} is not correlated with $u_{ikj}$, and treats {$Hineq_{ikjt-2}$, $Hineq_{ikjt-3}$, … } and {$\Delta Hineq_{ikjt-1}$, $\Delta Hineq_{ikjt-1}$,…} as instrumental variables of $\Delta Hineq_{ikjt-1}$. For the extended model, the instrumental variables of $\Delta Hineq_{ikjt-1}$ and assumptions are identical to the baseline model.

## 5. Empirical Results and Discussion

### 5.1. Baseline Results

The estimation results of Equation (1) are reported in Table 3. The sample size is 8370. Clustered robust standard errors are seen in parentheses, and ***, ** and * represent *p* < 0.01, *p* < 0.05 and *p* < 0.1, respectively. The results of the sys-GMM estimate reveal that the standardized coefficients are −0.10 for the population death rate, which is significant at the 1% significance level; −0.13 for the pregnant women death rate at the 1% significance level; −0.11 for the underweight newborn rate at the 1% significance level; −0.23 for the child malnutrition rate at the 1% significance level; and −0.09 for the infectious disease mortality at the 5% significance level. The empirical results reveal the negative causal impacts of ecological management on health inequality for population death rate, pregnant women death rate, underweight newborn rate, child malnutrition rate, and infectious disease mortality, on the assumption that individuals do not make efforts to improve their health status. We utilized ecological management lagged by 1 year to replace the level term of ecological management for the baseline model so that we could consider the impact of time delays of ecological management on regional health inequality. The estimated coefficients of ecological management lagged by 1 year are statistically significant for (1) population death rate; (2) pregnant women death rate; (3) underweight newborn rate; (4) children malnutrition rate; (5) infectious disease mortality, suggesting that the ecological management has a significant causal effect on regional heath inequality when individuals do not intend to make efforts to improve their health status. The findings are consistent with those in Table 3. The results of the Arellano–Bond test for autocorrelation of the first-differenced residuals and the Sargan–Hansen overidentification test suggest that the sys-GMM estimation results are valid in the baseline model and all the IVs included are valid.

### 5.2. Extended Results

After adding variables of the dietary, cultural circle, and education inequality in the baseline model, we can test whether ecological management also causes a decrease in Chinese health inequality when individuals try to improve their health, by leveraging a sys-GMM method. The results for the extended model in Equation (2) are reported in Table 4. The sample size is 8370. Clustered robust standard errors are seen in parentheses, and ***, ** and * represent *p* < 0.01, *p* < 0.05 and *p* < 0.1, respectively. The results of the sys-GMM method reveal that the coefficients are −0.10 and significant for the population death rate at the 5% significance level; −0.13 for pregnant women death rate at the 1% significance level; −0.12 for the underweight newborn rate at the 1% significance level; −0.30 for child malnutrition rate at the 1% significance level; and −0.13 for infectious disease mortality at the 1% significance level. The empirical results imply negative causal impacts of ecological management on health inequality for population death rate, pregnant women death rate, underweight newborn rate, child malnutrition rate, and infectious disease mortality—when individuals make efforts to improve their health status. Additionally, we substituted ecological management lagged by 1 year for ecological management in the extended model so that we could take account of the impact of time delays of ecological management on regional health inequality. The estimated coefficients of ecological management lagged by 1 year are also statistically significant for population death rate, pregnant women death rate, underweight newborn rate, children malnutrition rate, and infectious disease mortality. The results imply that ecological management has a significant causal effect on regional heath inequality when individuals make efforts to improve their health status. The findings are consistent with those in Table 4. The finding from this study is an extensive analysis of Health Field Concept theory by Lalonde [14].

Consistent with the baseline model, the results of the Arellano–Bond test for autocorrelation of the first-differenced residuals and the Sargan–Hansen overidentification test suggest that the sys-GMM estimation results are valid in the baseline model and all the IVs included are valid.

### 5.3. Heterogeneity

In this subsection, we focus on the heterogeneous impact of ecological management on regional health inequality. The results of the Chow test for regression on the subsamples in identical or different regions concerning the baseline model (Equation 1) are demonstrated in Table 5. Clustered robust standard errors are seen in parentheses, and ***, ** and * represent *p* < 0.01, *p* < 0.05 and *p* < 0.1, respectively. The subsamples of province pairs applied in models (1)–(5) are the province pairs lying in the identical region, and the province pairs in models (6)–(10) belong to different regions, respectively. The sample size for models (1)–(5) is 2682 and 5688 for models (6)–(10). The Chow test statistics are 0.92 (*p*-value= 0.5867), 4.59 (*p*-value = 0.0000), 5.47 (*p*-value = 0.0000), 7.50 (*p*-value = 0.0000), and 8.50 (*p*-value = 0.0000) for population death rate, pregnant women death rate, underweight newborn rate, child malnutrition rate, and infectious disease mortality, respectively.

Furthermore, the results of the Chow test for regression on the subsamples in identical or different regions for the extensive model (Equation 2) are demonstrated in Table 6. Clustered robust standard errors are seen in parentheses, and ***, ** and * represent *p* < 0.01, *p* < 0.05 and *p* < 0.1, respectively. The subsamples of province pairs applied in models (1)–(5) are the province pairs lying in the identical region, and the province pairs in models (6)–(10) belong to different regions, respectively. The sample size for models (1)–(5) is 2682 and 5688 for models (6)–(10). The Chow test statistics are 0.85 (*p*-value = 0.7191), 4.83 (*p*-value = 0.0000), 6.67 (*p*-value = 0.0000), 6.66 (*p*-value = 0.0000), and 7.58 (*p*-value = 0.0000) for population death rate, pregnant women death rate, underweight newborn rate, child malnutrition rate, and infectious disease mortality, respectively. In the extended model, the heterogeneity results (Table 6) are largely consistent with those in the baseline model.

Overall, the Chow test results for the baseline and extended models suggest that ecological management has significant heterogenous effects on regional health inequality for the last four health indicators; however, it is not significant for the population death rate. Specifically, the death rate among pregnant women, the underweight newborn rate, and the child malnutrition rate obtain an ‘ecological management divide’ both in the same region and across various regions; however, the alleviation of regional health inequality for ecological management is more significant and higher for the former subsamples. Additionally, without individuals’ efforts, the infectious disease mortality rate in the same region is statistically and economically significant, yet insignificant in differing regions; however, with individuals’ efforts, the infectious disease mortality rate in differing regions is statistically and economically significant, yet insignificant in the same region. The mechanism makes sense that individuals’ efforts help decrease infectious disease mortality in the same region, and then inequality in infectious disease mortality tends to shrink in different regions when the government intervenes to make efforts to improve the ecological environment.

## 6. Conclusions and Policy Implications

Regional health inequality is a significant issue in China [7] and globally [8]. This study focused on the causal effect of ecological management on regional health inequality across China. Our research entailed a health inequality analysis of paired provinces by adopting the Health Field Concept model and Effort–Circumstances theory. An entropy weight method of 31 Chinese provinces between 2001 and 2019 was performed to measure the ecological management level. The key findings from a series of empirical analyses of 8835 Chinese province pairs are as follows. Firstly, if individuals try to improve their health, the ecological management provided by authorities decreases regional health inequality across China. Secondly, if we relax the assumption that individuals and government make efforts simultaneously, ecological management significantly reduces regional health inequality.

The major conclusions and implications of this study are as listed below. First, we propose a framework to measure ecological management, which is a new perspective for assessing the level of ecological management. Compared to other assessment frameworks, our framework is based on the strategy adopted by the Chinese government. Our study’s indicators measuring ecological management are comprehensive and address the current limitations of ecological management. Quantifying the indicators is helpful for the central government to assist, guide, and supervise central and local officials in implementing ecological management.

Second, the bilateral approach accurately measures pure regional health inequality. Compared with the two traditional methods outlined above, the bilateral approach is practical and reliable for assessing pure regional health inequality and could be harnessed in future regional inequality analyses. Moreover, this approach is practical for policymakers in other countries to calculate pure regional health inequality or regional inequality in other aspects.

Third, the empirical analysis of ecological management used in this study—including the effort made by the government—is an extension of the Health Field Concept model and Effort–Circumstances theory, and could be further applied in the broader health economics and inequality fields. Thus, it validly extends the theory in health and inequality domains and provides new insights for policymakers.

Finally, yet importantly, the key findings from this study are that ecological management in China harms the corresponding regional health inequality in population death rate, the death rate among pregnant women, the underweight newborn rate, the child malnutrition rate, and infectious disease mortality. These empirical findings offer solid evidence that ecological management is effective for the government in decreasing regional health inequality. The finding may be relevant for other countries similar to China. Of these, the child malnutrition rate obtained the strongest marginal causal effect, followed by the death rate among pregnant women. Accordingly, if the government plans to reduce regional health inequality across China, it could be particularly significant for child malnutrition and death rates among pregnant women since the policy would be more beneficial for these groups than other health indicators.

Nonetheless, this study has some limitations that need to be further explored. Firstly, we used macro data to explore the effect of ecological management on regional health inequality. Further explorations of health inequality at the micro level are therefore vital to collect more indicators of human biology, environmental factors, lifestyle factors, and healthcare organization. Secondly, we provided strong evidence of the causal effect of ecological management on regional health inequality. However, the impacting pathways for the causal effect of ecological management are not addressed. Therefore, further research is required to explore the impact pathways of ecological management, such as moderating or mediating effects on inequality in population death rate, the pregnant women death rate, the underweight newborn rate, the child malnutrition rate, and infectious disease mortality.

## Figures and Tables

**Table 1:** Indicators and items to measure ecological management.

Indicators	Items
Water environment ^a^ (8 items)	Total wastewater discharge ^b^ (ten thousand tons)
	Cadmium Emissions ^a^ (kg)
	hexavalent chromium emission ^a^(kg)
	Lead emission ^a^ (kg)
	Mercury emission ^a^ (kg)
	Arsenic emissions ^a^ (kg)
	The chemical oxygen demand of wastewater ^a^ (ten tons)
	Petroleum discharge of wastewater ^a^ (tons)
Air environment ^a,d^ (4 items)	Total sulfur dioxide emissions ^a,d^ (ton)
	Nitrogen oxide emissions ^a,d^ (ten thousand tons)
	Carbon dioxide emissions ^b^ (ten thousand tons)
	Smoke (powder) dust emissions ^a,d^ (ten thousand tons)
Pollution treatment ^a,c^ (6 items)	The comprehensive utilization rate of industrial solid waste ^a^ (%)
	The urban sewage treatment rate ^a^ (%)
	Total sewage treatment capacity ^b^ (ten cubic/day)
	Centralized sewage treatment ^a,c^ (ten thousand tons)
	Sewage recycling amount ^c^ (ten thousand tons)
	Harmless disposal rate of household garbage ^a^ (%)
Green environment ^a,c^ (5 items)	The proportion of nature reserves in the jurisdiction area ^a^ (%)
	Urban green space area ^b^ (ten thousand hectares)
	Park green space area ^c^ (ten thousand hectares)
	The total wetland area of land area ^a^ (%)
	Forest coverage rate ^a^ (%)
Residential environment ^a^ (4 items)	Gas penetration rate ^a^ (%)
	Sanitary toilet penetration rate in rural areas ^a^ (%)
	Rural biogas digester gas production ^a^
	The water penetration rate ^b^ (%)
Soil environment ^a^ (3 items)	Domestic waste landfill amount ^a^ (ten thousand tons)
	Pesticide usage ^a^ (ten thousand tons)
	Paramount of agricultural fertilizer ^a^ (ten thousand tons)

Note: ^a^ Guo et al. [10]; ^b^ The indicators, most relevant to ecological management, are elaborated by the authors; ^c^ Chen and Shi [46]; ^d^ Brand and Missaoui [47].

**Table 2:** Definitions of variables.

Variables	Variable Symbols	Definition	Obs	Mean	Std. Dev.	Min	Max
Dependent variable	Population death rate	The absolute value of the logarithmic difference for the province pair’s health indicator, the death rate (‰)	8835	0.309	0.234	0	1.255
	Pregnant women death rate	The absolute value of the logarithmic difference for province pair’s health indicator, Pregnant death rate (per 100,000 persons)	8835	0.737	0.591	0	3.321
	Underweight newborn rate	The absolute value of the logarithmic difference for province pair’s health indicator, rate of the newborn baby with birth weight under 2500 g (%)	8835	0.361	0.294	0	1.568
	Child malnutrition rate	The absolute value of the logarithmic difference for province pair’s health indicator, rate of children under 5 years old with severe malnutrition (%)	8835	0.629	0.554	0	3.06
	Infectious disease mortality	The absolute value of the logarithmic difference for province pair’s health indicator, infectious disease mortality (per 100,000 persons)	8835	0.304	0.265	0	1.302
Key variable	Ecological Management	Province pair’s overall ecological management level, the sum of the ecological management between province pair	8835	0.755	0.138	0.377	1.334
Control variable	ABO	Nei’s gene distance of blood type ABO genes between province pair	8835	0.006	0.005	0	0.028
	Gm	Nei’s gene distance of immunoglobulin allotypes Gm gene between province pair	8835	0.181	0.203	0	1.165
	RGDP	GDP per capita: the absolute value of the logarithmic difference for province pair’s GDP, adjusted by CPI in 2001	8835	0.522	0.43	0	2.374
	POP	Provincial population: the absolute value of the Logarithmic difference for province pair’s permanent population at the end of year *t*	8835	0.961	0.775	0	3.589
	GSA ^a^	The absolute value of the logarithmic difference for province pair’s green space area in cities	8835	10.179	15.815	0	281.5
	Dist ^b^	Geographical distance for the province pair (logarithmic form)	8835	7.058	0.614	4.641	8.177
	Border	If province *i* is geographically adjacent to province *k*, border = 1, otherwise border = 0	8835	0.146	0.353	0	1
	region 1 ^c^	Region 1 = 1 if province pairs both lie in Eastern China	8835	0.118	0.323	0	1
	region 2	Region 2 = 1 if province pairs both lie in Central China	8835	0.06	0.238	0	1
	region 3	Region 3 = 1 if province pairs both lie in Western China	8835	0.142	0.349	0	1
	region 4	Region 4 = 1 if province pairs lie in Eastern China and Central China, respectively	8835	0.189	0.392	0	1
	region 5	Region 5 = 1 if province pairs lie in Eastern China and Western China, respectively	8835	0.284	0.451	0	1
	EXPEN	Health expenditure per capita: the absolute value of the logarithmic difference for province pair’s per capita healthcare expenditure, adjusted by CPI in 2001	8835	0.381	0.326	0	2.369
	URB	Hospital bed utilization: the ratio of bed utilization proportion of hospitals in province *i* in year *t* and beds utilization proportion of hospitals in province *k* in year *t*	8835	0.172	0.306	0	2.691
	POR	Patient outflow rate: the ratio of out-patient proportion in province *i* in year *t* and out-patient proportion in province *k* in year t	8835	0.014	0.028	0	0.283
	EDU	Education attainment: the ratio of average education attainment in province *i* in year *t* and average education attainment in province *k* in year *t*	8835	0.153	0.208	0	1.828
	Diet 1 = Jingjin	If province pairs are in the Jingjin diet culture circle, Jingjin = 1, or else Jingjin = 0	8835	0.002	0.046	0	1
	Diet 2 = Dongbei	If province pairs are in the Dongbei diet culture circle, Dongbei = 1, or else Dongbei = 0	8835	0.013	0.113	0	1
	Diet 3 = Zhongbei	If province pairs are in the Zhongbei diet culture circle, Zhongbei = 1, or else Zhongbei = 0	8835	0.022	0.145	0	1
	Diet 4 = Xibei	If province pairs are in the Xibei diet culture circle, Xibei = 1, or else Xibei = 0	8835	0.013	0.113	0	1
	Diet 5 = Huanghezhong	If province pairs are in the Huanghezhong diet culture circle, Huanghezhong = 1, or else Huanghezhong = 0	8835	0.032	0.177	0	1
	Diet 6 = Huanghexia	If province pairs are in the Huanghexia diet culture circle, Huanghexia = 1, or else Huanghexia = 0	8835	0.032	0.177	0	1
	Diet 7 = Changjiangzhong	If province pairs are in the Changjiangzhong diet culture circle, Changjiangzhong = 1, or else Changjiangzhong = 0	8835	0.006	0.08	0	1
	Diet 8 = Changjiangxia	If province pairs are in the Changjiangxia diet culture circle, Changjiangxia = 1, or else Changjiangxia = 0	8835	0.013	0.113	0	1
	Diet 9 = Dongnan	If province pairs are in the Dongnan diet culture circle, Dongnan = 1, or else Dongnan = 0	8835	0.045	0.208	0	1
	Diet 10 = Xinan	If province pairs are in the Xinan diet culture circle, Xinan = 1, or else Xinan = 0	8835	0.022	0.145	0	1
	Diet 11 = Qingzang	If province pairs are in the Qingzang diet culture circle, Qingzang = 1, or else Qingzang = 0	8835	0.022	0.145	0	1

Notes: ^a^ ln (green space area in cities +0.001), unit: 10,000 square meters. ^b^ The statistical description of the 18 yearly dummies is not included in Table 1. ^c^ The base group is ‘region 6 = 1 if province pairs lie in Central China and Western China, respectively’.

**Table 3:** Baseline results of sys-GMM method.

	(1) Population Death Rate	(2) Pregnant Women Death Rate	(3) Underweight Newborn Rate	(4) Child Malnutrition Rate	(5) Infectious Disease Mortality
	m1	m2	m3	m4	m5
Ecological management	−0.163 ***	−0.546 ***	−0.226 ***	−0.917 ***	−0.169 **
	(0.080)	(0.178)	(0.094)	(0.213)	(0.087)
Gm	0.446	0.875	0.029	−1.169	−1.171
	(0.708)	(4.842)	(1.072)	(3.837)	(1.482)
ABO	2.385	−26.741	−19.341	59.289	20.931
	(26.214)	(164.025)	(30.342)	(118.431)	(38.035)
RGDP	−0.065	−0.162	0.031	0.473 ***	−0.083
	(0.048)	(0.115)	(0.061)	(0.107)	(0.109)
POP	0.055	0.894 **	−0.468 ***	0.020	0.084
	(0.081)	(0.379)	(0.164)	(0.375)	(0.155)
GSA	−0.000 **	0.001 **	−0.000 *	−0.001 **	−0.000
	(0.000)	(0.000)	(0.000)	(0.001)	(0.000)
Dist	0.094	−1.627	0.319	0.567	−0.459
	(0.349)	(1.044)	(0.417)	(0.508)	(0.517)
border	0.281	−5.315 **	−0.010	0.504	−1.428
	(0.556)	(2.086)	(0.953)	(1.744)	(1.113)
region 1	0.121	−0.868	0.138	1.545	0.708
	(0.560)	(2.957)	(1.009)	(2.282)	(1.178)
region 2	−0.356	3.847	1.873	5.670	1.372
	(0.848)	(5.633)	(1.229)	(3.841)	(1.571)
region 3	0.296	1.826	0.000	1.522	0.607
	(0.720)	(1.506)	(0.509)	(1.520)	(0.621)
region 4	0.510	2.371	−0.880	−0.487	−2.214
	(0.676)	(3.823)	(1.036)	(2.466)	(1.404)
region 5	0.080	1.656	−0.132	1.506	−1.137
	(0.413)	(1.566)	(0.328)	(1.166)	(0.723)
EXPEN	0.012	0.060	0.026	−0.078 **	−0.086 ***
	(0.023)	(0.047)	(0.022)	(0.039)	(0.022)
URB	−0.083 ***	0.049	0.127 ***	−0.069	0.062 ***
	(0.020)	(0.066)	(0.022)	(0.049)	(0.024)
POR	−0.338 **	−0.880 **	−0.026	0.401	−0.224 *
	(0.156)	(0.396)	(0.210)	(0.293)	(0.127)
Death rate lagged for 1 year	−0.034 ***				
	(0.012)				
Pregnant death rate lagged for 1 year		0.006			
		(0.011)			
Newborn baby with weight less than 2500 g lagged for 1 year			−0.063 ***		
			(0.010)		
Children under 5 years old with severe malnutrition lagged for 1 year				0.027 **	
				(0.012)	
Infectious disease mortality lagged for 1 year					−0.092 ***
					(0.013)
Arellano–Bond test ^a^	0.0822	−1.4311	−1.1492	0.6048	−0.6812
(*p*-value)	(0.9345)	(0.1524)	(0.2505)	(0.5453)	(0.4958)
Sargan–Hansen test ^b^	2.6371	0.0914	0.2040	0.8838	0.1463
(*p*-value)	(0.2675)	(0.7623)	(0.6515)	(0.3472)	(0.7021)

Notes: ^a^ Arellano–Bond test for autocorrelation of the first-differenced residuals. ^b^ Sargan–Hansen test of the overidentifying restrictions.

**Table 4:** Extended results of sys-GMM method.

	(1) Population Death Rate	(2) Pregnant Women Death Rate	(3) Underweight Newborn Rate	(4) Child Malnutrition Rate	(5) Infectious Disease Mortality
	m1	m2	m3	m4	m5
Ecological management	−0.177 **	−0.536 ***	−0.265 ***	−1.191 ***	−0.246 ***
	(0.080)	(0.170)	(0.089)	(0.190)	(0.067)
Gm	−11.030	75.588	−2.932	−27.517	7.628
	(52.151)	(172.526)	(23.737)	(33.762)	(18.435)
ABO	0.000	0.000	0.000	0.000	0.000
	(.)	(.)	(.)	(.)	(.)
RGDP	−0.069	−0.079	−0.037	0.278 ***	−0.001
	(0.047)	(0.108)	(0.058)	(0.102)	(0.052)
POP	0.009	1.034 ***	0.248 **	0.918 ***	−0.130
	(0.105)	(0.219)	(0.111)	(0.208)	(0.094)
GSA	−0.000 *	0.001 **	−0.000	−0.001 **	−0.000
	(0.000)	(0.000)	(0.000)	(0.001)	(0.000)
Dist	−2.438	−8.513	11.950	3.653	−0.564
	(16.186)	(23.887)	(43.644)	(6.344)	(9.233)
border	−9.534	−15.600	26.668	−18.095	−5.744
	(48.764)	(48.331)	(116.390)	(14.319)	(25.531)
region 1	−15.123	30.785	32.258	−5.121	−1.543
	(68.951)	(43.940)	(132.892)	(15.289)	(40.739)
region 2	−10.466	7.722	−4.033	−11.623	−8.898
	(46.556)	(80.974)	(27.851)	(31.790)	(37.274)
region 3	7.139	−35.543	9.447	9.066	0.152
	(25.193)	(65.507)	(48.341)	(16.958)	(32.408)
region 4	8.741	−48.294	10.876	12.618	7.427
	(27.072)	(96.938)	(48.495)	(30.276)	(20.417)
region 5	1.173	−26.596	15.423	−8.076	0.305
	(10.403)	(61.015)	(59.279)	(6.930)	(9.753)
EXPEN	0.012	0.082 *	0.020	−0.080 **	−0.085 ***
	(0.023)	(0.045)	(0.021)	(0.038)	(0.019)
URB	−0.082 ***	0.054	0.120 ***	−0.087 *	0.059 **
	(0.019)	(0.065)	(0.021)	(0.048)	(0.023)
POR	−0.328 **	−0.869 **	−0.032	0.383	−0.226 *
	(0.157)	(0.387)	(0.207)	(0.289)	(0.122)
Edu	−0.248 ***	−0.136	−0.375 ***	0.432 ***	0.112 **
	(0.057)	(0.128)	(0.058)	(0.127)	(0.053)
Diet 1 = Jingji	0.000	0.000	0.000	0.000	0.000
	(.)	(.)	(.)	(.)	(.)
Diet 2 = Dongbei	13.002	4.037	33.515	−8.725	8.568
	(80.332)	(103.161)	(157.504)	(43.352)	(102.988)
Diet 3 = Zhongbei	−0.989	97.797	−82.290	−29.041	−14.665
	(42.124)	(140.519)	(329.945)	(41.408)	(42.471)
Diet 4 = Xibei	30.783	15.374	42.296	−6.593	22.711
	(156.241)	(152.167)	(180.150)	(29.289)	(124.564)
Diet 5 = Huanghezhong	−11.962	123.936	53.081	22.859	18.846
	(62.223)	(231.489)	(247.267)	(29.705)	(25.724)
Diet 6 = Huanghexia	5.226	45.789	−23.836	−19.987	−2.758
	(29.025)	(210.413)	(76.382)	(28.745)	(12.337)
Diet 7 = Changjiangzhong	0.000	−6.004	0.000	−4.488	0.000
	(.)	(178.462)	(.)	(63.599)	(.)
Diet 8 = Changjiangxia	17.089	−173.117	13.727	28.723	6.475
	(90.508)	(303.461)	(146.348)	(34.788)	(38.091)
Diet 9 = Dongnan	18.617	−13.894	0.122	1.303	−0.837
	(76.253)	(71.766)	(40.844)	(12.674)	(28.149)
Diet 10 = Xinan	10.926	2.473	−2.653	9.277	22.863
	(43.060)	(111.989)	(71.338)	(26.338)	(55.066)
Diet 11 = Qingzang	1.505	0.000	−16.670	0.000	−6.903
	(36.377)	(.)	(123.211)	(.)	(28.048)
Death rate lagged for 1 year	−0.039 ***				
	(0.011)				
Pregnant death rate lagged for 1 year		−0.042 ***			
		(0.010)			
Newborn baby with weight less than 2500 g lagged for 1 year			−0.081 ***		
			(0.010)		
Children under 5 years old with severe malnutrition lagged for 1 year				0.010	
				(0.011)	
Infectious disease mortality lagged for 1 year					−0.086 ***
					(0.011)
Arellano–Bond test ^a^	0.0196	−1.3787	−1.1630	0.6476	−0.6881
(*p*-value)	(0.9843)	(0.1680)	(0.2448)	(0.5173)	(0.4914)
Sargan–Hansen test ^b^	2.9109	0.0637	0.6448	0.9741	0.1616
(*p*-value)	(0.2333)	(0.8007)	(0.4220)	(0.3237)	(0.6877)

Notes: ^a^ Arellano–Bond test for autocorrelation of the first-differenced residuals. ^b^ Sargan–Hansen test of the overidentifying restrictions.

**Table 5:** Heterogenous regions: province pair in identical region vs. different regions (Baseline model).

	(1) Population Death Rate	(2) Pregnant Women Death Rate	(3) Underweight Newborn Rate	(4) Child Malnutrition Rate	(5) Infectious Disease Mortality	(1) Population Death Rate	(2) Pregnant Women Death Rate	(3) Underweight Newborn Rate	(4) Child Malnutrition Rate	(5) Infectious Disease Mortality
	Province Pairs in the Same Region					Province Pairs in Different Regions				
Ecological management	−0.182 *	−0.433 *	−0.328 ***	−0.827 ***	−0.244 ***	−0.122 *	−0.446 **	−0.160 *	−1.039 ***	−0.098
	(0.139)	(0.300)	(0.153)	(0.328)	(0.123)	(0.090)	(0.234)	(0.110)	(0.217)	(0.086)
Control ^a^	Yes	Yes	Yes	Yes	Yes	Yes	Yes	Yes	Yes	Yes

Notes: ^a^ The “Control” variables include human biology, environment, and healthcare organization.

**Table 6:** Heterogeneous region: province pair in identical region vs. different regions (extended model).

	(1) Population Death Rate	(2) Pregnant Women Death Rate	(3) Underweight Newborn Rate	(4) Child Malnutrition Rate	(5) Infectious Disease Mortality	(1) Population Death Rate	(2) Pregnant Women Death Rate	(3) Underweight Newborn Rate	(4) Child Malnutrition Rate	(5) Infectious Disease Mortality
Ecological management	−0.220 *	−0.500 **	−0.414 ***	−1.069 ***	−0.129	−0.112	−0.499 ***	−0.199 **	−1.149 ***	−0.116 *
	(0.148)	(0.302)	(0.149)	(0.319)	(0.110)	(0.091)	(0.209)	(0.107)	(0.226)	(0.088)
Control ^a^	Yes	Yes	Yes	Yes	Yes	Yes	Yes	Yes	Yes	Yes

Notes: ^a^ The “Control” variables include human biology, environment, lifestyle, and healthcare organization.

## Data Availability

The data that support the findings of this study can be downloaded on China National Bureau of Statistics, which is an open official website, and the provincial annual data are available at https://data.stats.gov.cn/easyquery.htm?cn=C01 (accessed on 20 December 2022).
